# Research and Technology Organizations as Super Intermediaries: A Conceptual Framework for Policy and a Case Study From Tanzania

**DOI:** 10.3389/frma.2021.691247

**Published:** 2021-06-17

**Authors:** Gussai H. Sheikheldin

**Affiliations:** Science, Technology and Innovation Policy Research Organization (STIPRO), Dar es Salaam, Tanzania

**Keywords:** research and technology organizations (RTOs), industrial development, research policy, triple helix (TH) model, innovation systems, Tanzania

## Abstract

Research and Technology Organizations (RTOs) have key roles in stories of national industrial development in many countries, and in various contexts they have transformed according to changes in their surrounding economic and policy environments. This paper proposes a conceptual framework of ‘RTOs as super intermediaries’ as they play multiple intermediary roles in the triple helix (government, research and industry), the overlap of industrial policy and research policy, and research-industry frontiers. The framework helps in understanding and advancing the role of RTOs in industrial development, particularly in developing countries. For a case study, the paper showcases research in Tanzania that explored possibilities of revamping RTOs and whether investing in them would help in spurring Tanzania's industrial development. Through key informant interviews and systemic literature review, a case study on the challenges and opportunities of RTOs was designed to examine their role and potential in industrial development and technology innovation processes. The study findings were overall in-line with two main lenses of inquiry: 1) that for RTOs to play their key roles in Tanzania, industrial policies shaped by the command economy era before the 1990s need to be reviewed and modified; and 2) that more investment in revamping RTOs will take place if policymaking processes acknowledge RTOs as super intermediaries. To organize policy lessons drawn, a multi-level policy map—micro, meso and macro—was utilized as an analytical tool.

## Introduction

Industrial, Research and Technology Organizations have various names and classifications across countries, but a consensus has been reached in referring to them as RTOs.^1^ Some RTOs around the world have been key contributors to national industrialization and in shaping their industrial policies (Nath and Mrinalini 2000; United Nations’ Economic Commission for Africa, 2016). For example, RTOs in newly industrialized economies (NIEs) of Asia combined technological innovation and enterprise incubation to conceive and diffuse key products and systems in their countries’ industries and markets (Wolff 1999; Ash et al., 2006). Lall and Pietrobelli (2005) argue that, for many African countries RTOs play a key role as contributors and indicators for either positive or negative trajectories of national technology systems. Many RTOs are parastatals, meaning they are entities where the state is either the owner or the main shareholder but the government does not directly manage their operations. RTOs focus on research and development (R&D) with the purpose of playing a critical intermediary role between applied research in science, technology and innovation (STI), industries, markets and related policies according to national development agenda. Typically, they run as research centres hosting researchers, technologists, and others, with a view to improve technological capabilities in chosen sectors through finding and materializing technological and systems solutions to industrial and development problems, adapting and modifying foreign technologies for local/national contexts or inventing new technological products or services to meet local demands, and providing technical and policy assistance to industries and governments (acting as consultancies and/or think tanks). The European Association of Research and Technology Organizations [European Association of Research and Technology Organization (EARTO), 2015] defines RTOs’ core mission as, “to harness science and technology in the service of innovation, to improve quality of life and build economic competitiveness” (2015, 3). The role that RTOs play is unique; Giannopoulou et al. (2019) found that RTOs play different (but complimentary) roles in national innovation systems than academic institutions (universities) and that investing in RTOs renders different outcomes as well.

Worldwide, RTOs have gone through various transformations, with many due to the changes in their national/regional contexts and enabling environments. Sharif and Baark (2011) observed that RTOs in Europe and Asia were undergoing transitions due to key shifts in the environment within which they operate; particularly the authors highlighted "the increasing pressure to commercialise research outputs and the internationalisation of the research endeavor, providing new opportunities for both funding and transfer of outputs.” (p. 1). Preissl (2006) showed that RTOs in Europe were already changing their modes of operation as national economies shift towards service economies. Rincón Díaz and Albors Garrigós (2017) discuss that while RTOs in regions of Spain are going through institutional reforms due to economic changes (such as austerity policies that limit resources), "technological policy must consider the characteristics of each region" to make the changes efficient and realistic (p. 180). Changes to RTOs are reportedly connected to how policymakers perceive or understand their role in the bigger picture—when models of RTOs are persuading, they capture more policymakers’ attention (Albors-Garrigos, Zabaleta and Ganzarain 2010).

This paper presents a study that aims to inform policy, planning and further research about the role of RTOs, in support of industrialization and STI agenda, especially in developing countries. Through literature review and conceptual tools—tempered with fieldwork familiarity—a conceptual framework is proposed to understand the role of RTOs in industrial development and STI advancement as well as guide policy efforts to improve their capabilities and orientations. Using the framework as a lens of inquiry, the paper then presents a case study of RTOs in Tanzania, based on a research that aimed to identify policy challenges and opportunities of RTOs’ reform in the country.

In Tanzania, parastatals proliferated in the 1970s to build financial, agricultural, infrastructure, manufacturing and service industries. Their broad and varied experiences have been amply studied (Loxley and Saul 1975; Coulson 1982; World Bank 1988; Bongenaar and Szirmai 2000; Oyelaran-Oyeyinka and Sampath 2007; Temu and Due 2000). Decades later, most of them were either decommissioned or privatized while a few remain as they were. As R&D parastatals, RTOs in Tanzania are called ‘public technology intermediaries’ (URT 2015a; Diyamett and Risha 2015). According to the Tanzania industrial competitiveness report 2015, most of its RTOs remain active to date but with low productivity and many challenges. Before that, Tanzania has gone through various stages and challenges to industrialization since political independence, from import substitution in the early years of independence, to command economy policies from the late 1960s to the early 1980s, fostering a slow but steady industrial progress between 1975 and 1986, a period in which RTOs were quite active (also a period that witnessed many setbacks due to exogenous political and economic circumstances, such as the Kagera war, severe droughts, and global economic crises unfavourable to local industrialization), to structural adjustment policies between 1986 and 1995 that failed to increase industrialization, to the current period of prioritizing industrialization through national planning under mixed-economy policies (Wangwe et al., 2014; Morrissey and Leyaro 2015; Msame and Wangwe 2016). A summarized history of Tanzania’s periods of industrial development and guiding policies is in Table 1.

**TABLE 1:** Summary of history of industrial development since independence in Tanzania^a^.

Period	Main industrial features	Notes
1961 to 1967	Import substitution policy with little state participation	—
1967 to 1975	The arusha declaration established an era of command economy (in planning and execution). Means of production were nationalized and wealth distribution centralized. The state owned more industry and controlled foreign investment flows	National scientific research council (NSRC - UTAFITI) and small industries development organization (SIDO) were established in this period
1975 to 1986	A basic industrial strategy (BIS) was put together to achieve economic growth through local resources. Slow but steady industrial progress took place over this period, and national R&D institutions—such as PTIs - were relatively productive. Practices of price controls, import licenses and exchange rate overvaluation were not conducive to rapid industrialization	Most research and technology organizations (RTOs) were established in this period: TIRDO, TEMDO, CAMARTEC and COSTECH.
		Several external circumstances hindered the BIS performance, such as global economic crises of the 1970s, the kagera war, shifting terms of trade, severe droughts and the collapse of the first east african community
1986 to 1995	The economic recovery program (ERP) tried to redress imbalances that came with adopting a package of structural adjustment pushed by international main multilateral donors, as ways of stimulating growth and increasing industrialization. A period of privatization and trade liberalization ensued, but generally did not lead to increased industrialization; rather the opposite	When privatization and trade liberalization were prescribed to Tanzania, through the structural adjustment program, it was understood that a necessary phase of stagnation might happen as a result. However that phase stretched longer than prophesized
1995 to 2015	Privatization of industries was followed by measures to create incentives for private investment and industries (foreign and local). Additionally, a sustainable industry development policy was adopted in pursuit of sustainable industrial development—i.e., increasing employment and economic growth, reach equitable development, and mix import substitution with export promotion	“The most dynamic [industrial] subsectors in terms of output growth, export growth, production innovation, and product diversity are food products, plastic and rubber, chemicals, basic metal work, and non-metallic mineral products” [Msame and Wangwe, (2016), 155]
2016 to 2020	The 2nd national development plan (FYDP-II) prioritizes industrialization and enhancing human capital capacity and emphasizes the use of STI for that purpose. Tanzania seeks to usher a new era with intensified use of STI in development agenda and aiming to reach a semi-industrialized economy status by 2025	FYDP-II highlights some RTOs to be involved in flagship projects, but without focus on RTOs themselves

a Sources: Msame and Wangwe 2016; Morrissey and Leyaro 2015; Wangwe et al., 2014; URT, Ministry of Finance and Planning, 2016.

For the last decade, Tanzania has been planning and working to usher industrialization as a means of economic development (URT, Ministry of Industry and Trade, 2011; URT, Ministry of Finance and Planning, 2016). It has maintained a high level of economic stability and reasonable growth over the past decade, receiving recognition among the growing economies of Africa. Yet there are some qualifiers to consider, such as that the country’s economic growth seems less significant when we adjust it by considering population growth, becoming not highly above the average growth rate for sub-Saharan Africa (Page 2016). Additionally, decent-wage jobs that offer employment security have not increased in concert with overall growth, while the country has been seeking to productive industries to address development and employment challenges more effectively (World Bank 2014). The industrial sector—including manufacturing, agro-industry and skilled tradable services—has been largely absent from the Tanzania recent economic growth story, with significant long-term consequences. The industrial sector is capable of absorbing large numbers of skilled labour, thereby increasing employment and overall purchasing power in the economy. Aspects of supportive infrastructure in the country make conditions more challenging, such as energy and roads (Newman et al., 2016; United Republic of Tanzania 2017). Additionally, more than 70% of Tanzania’s private sector is in the informal economy, painting a private sector that is dominated by lower skills and limited access to finance and technology transfer (URT 2015b). Tanzanian small-and-medium enterprises (SMEs), and their clusters, do not yet have the requisite industrial capacity (Musonda 2007), and they must have it to industrialize the country’s economy. Overall, the big picture says that recent records of economic growth have not yet rendered proportional genuine economic development. On the other hand, Tanzania’s contemporary national planning aims to graduate to a middle-income, semi-industrial economy by 2025. As a strategy for achieving that goal, its 5-years national development plan 2016/17–2020/21 (FYDP-II) emphasizes industrialization and human development as a twin priority and highlights the fostering and utilization of science, technology and innovation (STI) for that purpose.

After this introductory section of the paper, a conceptual framework for understanding the role of RTOs is proposed. Following that is a section describing the case study design (including methodology) of Tanzanian RTOs. Findings of the study are then organized using a multi-level policy map (part of the study design) in addition to summarizing brief comparative cases from outside Tanzania. Discussion of the findings follows, and the final section provides conclusions and broad recommendations for policy reform.

## Research and Technology Organizations as Super Intermediaries: Triple Helix, Policy and Consultancy

**FIGURE 1:**
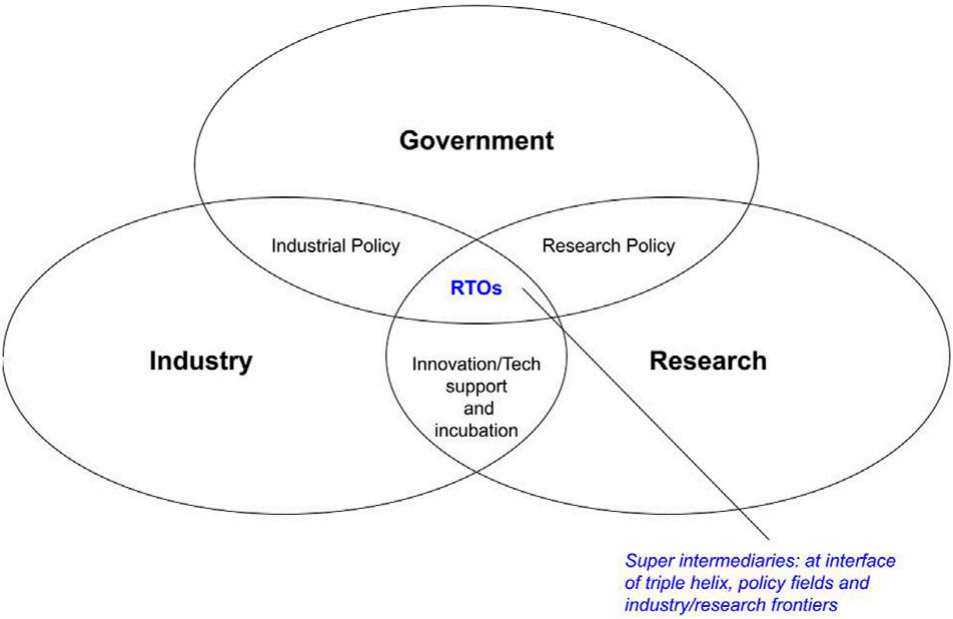
Conceptual framework of RTOs as super intermediaries.

This paper proposes a conceptual framework that sees RTOs as super intermediaries—meaning that they play multi-faceted intermediary roles, in modern societies, in systems that combine major actors such as government, industry and research circles (the triple helix model) as well as key policy fields such as industrial policy and research policy (Figure 1). In other words, RTOs are no ordinary intermediaries, and their importance should be duly heightened. In the triple helix, RTOs are situated at the interface of that model, as they interact extensively with the three components of the helix and also perform functions that are typically within the “specialization” of each component—a feat rarely witnessed in other organizations. In the policy arena, the overlap between industrial policy and research policy is one of the most critical areas in a country’s national innovation/technology system, and RTOs epitomize that overlap, not only in their role as key implementers of such policies but also as contributors to their making, since RTOs also function as policy advisors and think tanks in both policy fields. Finally, at the interface of research and industry RTOs are there as consultancy and technical support providers to industrial firms, university researchers, training programs, etc. In the big picture, RTOs are uniquely situated to play key roles in spurring industrial development and innovation.

Understanding their place as super intermediaries, we can see RTOs as deserving of serious attention—especially from policymakers and policymaking processes—whenever a country’s industrial and STI development plans are discussed, and whenever such issues are studied. In developing countries, particularly ones that are endeavoring to soon become industrialized or semi-industrialized, special attention must be paid to RTOs under that orientation—their needs, their capacities, and their assigned functions in national development plans.

The proposed conceptual framework is supported by theoretical and empirical literature from three overlapping fields of study: industrial development, research policy and innovation systems—all of which have something relevant to say about RTOs. Recent literature on industrial development suggests that policies can make or break successful stories of industrialization around the world. In a 2016 volume, comprised of various industrial development comparative case studies, the editors relayed that, “comparative results from (Africa and Asia) case studies suggest that policy choices are largely responsible for the differences in industrialization outcomes” (Newman et al., 2016, 20). Furthermore, a 2016 report by the United Nations’ Economic Commission for Africa asserted that historical evidence points towards ‘transformative industrial policy for Africa’ as a main engine for economic development. Oqubay (2016) also reads the history of NIEs to extract, in no vague terms, how industrial policies were key to their industrialization. The above-mentioned studies do not fail to mention the significant role of RTOs, as they provide technical support, training, and incubation to two important actors: state-owned enterprises (SOEs) and SMEs, in addition to providing policymakers with sufficient industrial information and technical advice about key sectors and trends. Additionally, global comparative studies on RTOs confirm that their efficacy lies in being effective intermediaries between R&D and industry, with actionable accuracy being more important than classical quantitative indicators of performance (Nath and Mrinalini 2000; 2008).

In the research policy literature, it has become an axiom that continuous research is needed to inform sound research policy—particularly STI policy. The importance of using research to influence public policy, including research policy itself, invoked fields such as ‘science of science policy’ and ‘building science systems’ (Fealing et al., 2011; Eboh 2014; Diyamett, Makundi and Sheikheldin 2019; Hanlin et al., 2021). In these fields RTOs have been studied to explore their critical role, one that made Hanlin et al. (2018) and Chataway et al. (2019) call some RTOs ‘boundary organizations’—i.e., placed at the boundaries where innovation, science and policy actors meet (i.e., intermediaries).

In innovation systems’ studies, it is well-established that RTOs ‘have a pivotal role to play in creating national innovation systems’ (Nath and Mrinalini 2008, 37), or national technology systems (Lall and Pietrobelli 2005), especially in developing countries where they have been active at the interface of research and production. The difference between innovation and technology systems here is only contextual, with the latter concept arguably evolving from a critical assessment of two concepts: national innovation systems (Lundvall 1992) and national industrial systems (Pietrobelli 2001). “Since the bulk of technological activity in (developing countries) concerns the absorption and improvement of existing technologies rather than innovation at the Frontier, we prefer to use the term ‘national technology system’ in developing countries rather than ‘national innovation system” (Lall and Pietrobelli 2005, 313). Developing countries find themselves in need to hone conventional industrial development practices such as technology transfer, basic R&D (as opposed to Frontier R&D), reverse engineering, etc. which are not necessarily ‘innovative’ per the standard definition but can increase industrial and technological outputs and capabilities. Yet, it is also possible to understand that national innovation systems do not necessarily always speak about innovation at the Frontier, but rather what is ‘Frontier’ at a specific context; RTOs have been known to sometimes ‘innovate’, in that sense, at national/regional levels in developing societies. Additionally, in both industrialized and developing countries, and due to their significant role in knowledge brokering and spurring innovation through multiple tasks in the national innovation systems, some RTOs are classified as ‘innovation intermediaries’ among other actors (Howells 2006; Kilelu et al., 2011).

All in all, the proposed conceptual framework of ‘RTOs as super intermediaries’ stands on justified grounds.

## Revitalizing Research and Technology Organizations in Tanzania: Study Design

This study was designed for policy learning and of possibilities, using historical case study strategy. Through constructing a narrative from key informants’ feedback and systemic literature review, lessons for policy could then be relayed through a format of lessons and recommendations. Key informants that were targeted for this study are senior personnel in RTOs and relevant institutions, such as ministries, universities and RTOs from other countries of relevance. As holders of intimate knowledge on the topic they would not only articulate the challenges and issues clearly, as insiders, but also provide informed opinions about opportunities and what could be changed in terms of policies to make opportunities within reach. The role of the researcher consisted of asking key questions, collecting responses, comparing and balancing with existing relevant literature, and synthesizing findings, to follow by discussion and lessons.

The research question for this study was: What are the policy barriers and opportunities, surrounding RTOs in Tanzania, that influence their role as super intermediaries?

The two guiding lenses of inquiry, or broad motivations for this study, or the approach that guided the study, were:

1) that the current challenges that RTOs face can be traced down to two constraints: regulations and revenues, and that these constraints are an institutional/policy legacy, from their early years of establishment, during the command economy era in Tanzania, when the state dominated economic sectors and intervened heavily in the market. When the shift towards a mixed-economy orientation happened in Tanzania, throughout the 1990s and into the new millennium, RTOs were not restructured to suit the new context; and
2) that more investment in revamping RTOs will take place if policymaking processes acknowledge RTOs as key players on multiple fronts (i.e., super intermediaries).

The first lens was based on critical learning and understanding, from the author’s previous and present research and work in Tanzania^2^ as well as from its history of political economy and industrial development (Loxley and Saul 1975; Coulson 1982; Morrissey and Leyaro 2015). The second lens was based on the conceptual framework developed from the literature review conducted for this study (and later improved through field observations).

### Multi-Level Policy Map

**FIGURE 2:**
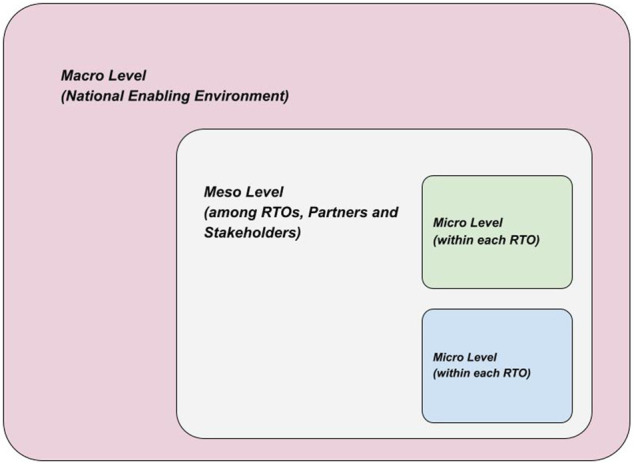
Multi-level Policy Map. *source of diagram idea: Van Notten (2006). Remade by author for new context.

The design of the study also aimed to make policy aspects well organized. For that, a multi-level policy map was chosen as a supporting analytical tool (for analysing findings): macro, meso, and micro policy levels. These levels are also called levels of institution-based scenarios (Van Notten 2006). A multi-level policy map designates levels that explored policies targeted in the big institutional/national picture (Figure 2). The rationale for using this multi-level policy map is that, firstly, RTOs operate under a similar national environment (the enabling or contextual environment) which reflects into common challenges that require attention at the macro level. Secondly, there are also challenges (and opportunities) that are more-or-less common among the RTOs but are neither contingent on the enabling environment nor on the organization-level practices. Such challenges relate to the nature of relations among actors in a sector to which all these RTOs belong in one way or another including their networks of communication and collaboration, and how much they know about each other and support each other (or compete with each other), a situation that calls for responses at a collective level between such actors but not necessarily in the form of macro/government intervention—that is the meso level (or the transactional environment). Thirdly, there are challenges that are particular to the organizational (micro) level, at which each RTO is expected to resolve internally. Additionally, in synthesizing policy ideas, the study aimed to make sure to align its content with national policy directives of the country where the study took place, in order to be relevant to context and limitations.

### Methodology

Historical case study strategy was deemed a fitting methodological approach to the study (Verschuren 2003; Bennett 2004). According to Verschuren (2003), a case study strategy is characterised by “looking at only a few strategically selected cases, observed in their natural context in an open-ended way, explicitly avoiding tunnel vision, making use of analytical comparison of cases or sub-cases, and it aimed at description and explanation of complex and entangled group attributes, patterns, structures or processes” (p.138). In order to learn lessons for the sake of informing policy, given that policy deals with complex factors entangled in real-world problems, a qualitative case study approach made more sense as a methodological/design choice for this study, and most of the data/information gathered was qualitative. RTOs are limited in numbers and are varied in size, specialization and structure in each country, therefore could not be quantitatively compared to each other or to similar organizations in other countries. Additionally, given that the research question and lens of inquiry are policy/governance oriented, with insight to be sought about different levels of policies in long term and without well-kept records of performance indicators in the country, a quantitative approach would have been too shallow, if not unattainable.

The study used sets of cases and comparative cases to build intelligence through two main research tools: key informant interviews and systemic literature review. The case Tanzanian RTOs were: COSTECH (Tanzania Commission for Science & Technology), TIRDO (Tanzania Industrial Research and Development Organization (TIRDO), 2011), SIDO (Small Industries Development Organization (SIDO), 2014), CAMARTEC (Centre for Agricultural Mechanization and Rural Technology) and TEMDO (Tanzania Engineering and Manufacturing Design Organization (TEMDO), 2011). All these organizations are RTOs with different yet overlapping functions within the Tanzanian state apparatus that promote R&D, industrial support and technology transfer and innovation (URT 1973; 1979; 1980; 1981; 1986). While COSTECH is mainly a science-granting council^3^ that supports and manages scientific research nationwide, it also has its own technology transfer and R&D activities.^4^ SIDO focuses more on supporting SMEs with industrial orientation to acquire and develop technological capabilities and resources to survive business bottlenecks, but it also conducts its own R&D in the process and collaborates with the other RTOs on R&D projects. TIRDO, CAMARTEC and TEMDO are mainly applied research and R&D focused, easily fulfilling the orthodox RTO definition. Table 2 provides summaries of their profiles.

**TABLE 2:** Research Participant Organizations (RTO case studies)^a^.

	Organization	Brief description	Established since…	Size	Notes
—	**COSTECH—Commission for science and technology**	Mandate: Principal advisory organ of the government on the use of science and technology for national development. Promotes and coordinates research for STI improvement as well as popularizes STI in society. Represents the state in bilateral and multilateral national STI programs. Reports to ministry of education, science and technology	1986 (1972)	Nationwide; HQ in dar es salaam plus one branch in zanzibar. 22 technical staff total (10 PhDs, 23 master’s)	Successor of the national scientific research council (NSRC - UTAFITI) that was established in 1972
—	**TIRDO—Tanzania industrial research and development organization**	Mandate: To undertake applied research which leads to industrial utilization of local materials, and support industry in technology transfer and technical services. Goal is to become a leading industrial research hub and make tanzanian industries become locally and internationally competitive. Reports to ministry of industry and trade	1979	Nationwide, with only one premises (HQ) in dar es salaam. Estate is large but not fully utilized. 78 workers total, 39 technical staff (8 PhD, 14 master’s, 8 advanced technical specialization, and the rest with basic technical training)	Viewed as technically the flagship of R&D RTOs in Tanzania, since it was the first with a clear mandate for specific industrial R&D
—	**SIDO—Small industries development organization**	Mandate: To create, promote and sustain innovative entrepreneurial base by providing SMEs with technical services, training, market intelligence, and business incubation. Reports to ministry of industry	1973	Nationwide; 21 offices 21 branches/regional offices which are located in each region around the country. The largest size among the cases. 401 workers total (including 40 master’s, 80 advanced technical specialisation, and others with basic technical training)	There is little in-house R&D, but a lot of collaboration with the other RTOs to disseminate their R&D results to small businesses adoption
—	**CAMARTEC—Centre for agricultural mechanization and rural technology**	Mandate: To function as an innovation centre for testing and building agricultural machinery and rural technology, disseminate improved technologies for agricultural and rural development, and support small enterprises that embark on innovating and marketing agricultural or rural technological products. Reports to ministry of industry	1981	Nationwide; two premises: HQ in arusha and nzega branch in tabora region. 72 technical staff (one PhD, 4 masters’, 10 advanced technical specialisation, and the rest with basic technical training)	The youngest and most specific mandate among the RTOs
—	**TEMDO—Tanzania engineering & manufacturing design organization**	Mandate: To research, develop and transfer plants and equipment for commercial manufacturing and deliver competitive engineering manufacturing knowhow and R&D services to the industrial sector. Reports to ministry of industry	1980	Nationwide presence; one office (HQs) in arusha. 31 technical staff (7 master’s degree; 9 advanced technical specialisation, and the rest with basic technical training)	In its early days, TEMDO led a few important national projects of technological ambition

a Table cumulated by the author using data from multiple sources, but Mainly Diyamett and Risha (2015) and the Industrial Competitiveness Report, 2015.

A guideline was designed to lead semi-structured interviews with selected key informants. About 35 Interviews were conducted, between April and June of 2016, mostly in-person. Guided tours around RTO facilities—to aid perspective on size, activities and capabilities—were provided to the researcher, as well as some prepared presentations (by key informants).^5^ Other communications and meetings took place in Kenya and Malaysia, in informal settings, with relevant personnel (such as senior personnel with SIRIM and the Malaysian ministry of STI) and included relevant public information, guided tours, and published literature. For data analysis, a qualitative research software (Nvivo v.11) was used.

## Findings

### Tanzanian Research and Technology Organizations Challenges and Opportunities

In this section, grouped and similar statements (consensus statements) from the key informant interviews are summarized and grouped according to the analytical tool—‘micro, meso and macro’ policy map—in Table 3. Another classification of statements follows the content of statements: constraints, observations and suggestions. While Table 3 provides the summary, some elaboration is provided below.

**TABLE 3:** consensus statements from key informants (organized to analytical tool: multi-level policy map).

Micro	Meso	Macro
*Constraints*	*Constraints*	*Constraints*
Being almost exclusively state-funded, RTOs face tight government budget constraints and fluctuating political support. As a result, they often receive less budget support than officially promised by the state	RTOs are not successful at attracting and retaining enough highly skilled individuals who can lead and manage innovative R&D and industrial support projects	Due to being commissioned as public-sector institutions RTOs are not able, by law, to commercialize their technological inventions/innovations (i.e. have in-house commercial mass-production and sale)
*Observations*	*Observations*	*Observations*
RTOs are already involved in some revenue-generating activities. Additionally, some RTOs join collaborative projects with national agencies that take care of the project’s budget including the RTOs’ activities’ costs. However, these activities contribute minimally and inconsistently to their budgets	The scope and breadth of research collaborations that exist on paper—in forms of MOUs, networks and bilateral/multilateral agreements—is not reflected in reality. Officially there are many arrangements and mechanisms in place for collaboration among RTOs, but in reality they work in siloes	RTOs do not have strategies of dissemination of ready-for-market innovations
*—*	RTOs seem to have a general misunderstanding that their performance is measured towards producing novel technologies, but as intermediary organizations for industrial support they are mainly expected to do ‘adaptation work,’ i.e. adapting technologies from various sources to local use	Policy orientation under command economy had implications such as that RTOs dealt mostly with other commercial parastatals. With the advent of mixed economy policies, however, RTOs had to interact with broader industries according to market rules. Policies and practices of RTOs remained the same while Tanzania changed economic models
*Suggestions*	*Suggestions*	*Suggestions*
Improve HR capacity by hiring more skilled staff, upgrading skills of existing staff, and giving them satisfying packages	RTOs should work together and communicate much more and should strengthen linkages with equivalent organizations in neighbouring countries	Acts of parliament governing RTOs (each and all) should be amended/updated
Improve the management systems of RTOs to optimize use of human, capital and financial resources in projects	Harmonize skills between RTOs and universities, so that there can be a horizontal two-way movement of qualified personnel	All RTOs should be managed under one umbrella, instead of running under various ministries as they are now
Regulations to increase practical programs such as incubators	RTOs’ clients should be empowered by measures such as including industry members in BODs	*—*
Improvement of infrastructure: Finish unfinished buildings in RTO premises, renew machine shops with modern equipment, and make laboratories certified	*—*	*—*

Three major constraints were mentioned consistently by personnel from Tanzanian RTOs. The first is revenue constraints: being almost exclusively state-funded, RTOs face tight government budget constraints and fluctuating political support. As a result, they often receive less budget support than officially promised by the state. In addition, RTOs have little exposure and access to other reliable sources of funding, for which they must compete against entities with larger research capacities such as universities. The second is regulatory constraints: RTO personnel claimed that, since commissioned as public-sector institutions they are not able, by law, to commercialize their technological inventions/innovations (i.e., have in-house commercial mass-production and sale). They said that once design prototypes are finalized, tested and proven ready for public use, RTOs are expected to wait until they can handover their technologies to other entities to commercialize them (such as private enterprises, capable NGOs, commercial parastatals, etc., ), but such scenario rarely happens. The third constraint is human resource capacity: for various reasons, RTOs are not successful at attracting and retaining enough highly skilled individuals who can lead and manage innovative R&D and industrial support projects. Apart from a small number of qualified personnel, RTOs are often unable to compete with universities, international agencies and big companies for the limited pool of relevant talents in Tanzania. It is difficult to study the human capacities of RTOs in detail since the turnover of qualified personnel is relatively high.

A majority of RTO respondents consistently mentioned these constraints with variation. If we look at one case, for example, TIRDO, we find that conditions that were generally described in the year 2000 about the performance of the RTO, in a detailed study (Bongenaar and Szirmai 2000), have not changed much:

“(The) study of TIRDO examined 12 of the 25 technology projects undertaken during 1979–1996…. The authors found that most projects were undertaken at the initiative of TIRDO staff rather than at the request of industry. Project evaluation did not look in depth at its technical or economic desirability for the economy or at its environmental aspects. The original technology on which projects were based was imported and mostly over five years old. Success was defined by the technical objectives of the staff rather than by application in industry or commercial success … Once developed, marketing of the technologies to potential users was weak … Despite its potential role in supporting, stimulating and producing industrial technology, (TIRDO) has not so far been able to link itself to industry, identify industrial needs or provide new technologies.” (Lall and Pietrobelli 2005, 332).

Respondents from COSTECH challenged the claim of the second constraint (regulatory constraints about generating own revenues). They argued that the legal acts that govern RTOs do not necessarily prohibit them from engaging in revenue-generating activities. Rather, RTOs have some flexibility for limited (and justified) mass production. Additionally, RTOs still can create spin-off enterprises (that can be fully fledged for-profit, the revenues of which can be partly paid to the RTOs as host/parent organizations) and generate revenue through patents and limited shares of returns from graduated incubatees; they also can enter into agreements with industries that take-up their technologies to get some royalties from commercial sales. As for patents, respondents from RTOs acknowledged that, to date, they registered no patents for any of their multiple technological innovations. Therefore, arguments about whether RTOs can create spin-offs, incubate systemically and utilize patents remain largely theoretical to date.

The Tanzanian national development guiding documents, such as FYDP-II, IIDS 2025 and the Tanzania Development Vision 2025, generally acknowledge RTOs as instruments for devising and modifying technologies that then are to be parts of bigger flagship projects and sectoral plans. RTOs are expected to be chartered with tasks of actualizing a package of policies of promoting industrialization. The FYDP-II states that it is intending to approach industrialization in a ‘business unusual’ manner, which implies that there will be ‘fundamental restructuring and repositioning in government undertakings’ (FYDP-II 2016; ii). RTOs had good reasons to be optimistic about the FYDP-II, as expressed by study respondents. Yet, the plan itself does not have a strategy of revamping RTOs, except for assigning some partial tasks to some of them. Yet, lessons from the study tell that RTOs need to revamp in order to do their job.

As for observations, key informant respondents provided commentaries and statements that showed keen readings into aspects of the situation of RTOs. One observation is that, currently, RTOs are already involved in some revenue-generating activities, in the form of technical consultancies, training workshops, rental of some of their property space, and paid fabrications (by request) to some clients. Additionally, for periods, some RTOs join collaborative projects with national agencies that take care of the project’s budget including the RTOs’ activities costs. However, these activities contribute minimally and inconsistently to their budgets.

Another observation is the scope and breadth of research collaborations that exist on paper—in forms of MOUs, networks and bilateral/multilateral agreements—but are not activated for one reason or another. Although officially there are many arrangements and mechanisms in place for collaboration among RTOs, respondents acknowledged that their RTOs generally work in siloes. Another observation is that these RTOs do not have strategies of dissemination of ready-for-market innovations. There is a common (and old) assumption that good prototypes will eventually reach local industries, for commercialization, in one way or another. This assumption results in the absence of diffusion strategies.^6^ Along the same observation, interviews with key informants outside RTOs argued that RTOs seem to have a general misunderstanding that their performance is measured towards producing novel technologies, which are good things to produce but not their main function. They argue that, as intermediary organizations for industrial support, RTOs are mainly expected to do ‘adaptation work,’ i.e., adapting technologies from various sources to local use and facilitating their adoption in society and the economy.^7^

A critical observation, by some RTO respondents, was directly related to this study’s lens of inquiry. Interviews and the relevant literature revealed that indeed, historical changes can explain much of the current constraints, and that RTOs themselves are aware of that.^8^ In its 2011–2016 strategic plan, TIRDO says:

“From 1980s to date, Tanzania has undergone various political, social and economical reforms and strategic changes. This includes the transformation from the state-controlled economy to a quasi-mixed economy and fundamental changes in national policies and strategies, which included the liberalization of the economy and privatization of industries. For instance, from the 1970s up to 1990s the majority of the industries were state owned. Currently, about 97% of industries in Tanzania are privately owned while only about 2% are publicly owned.” (p.3).

Policy orientation under command economy had implications such as that RTOs dealt mostly with other commercial parastatals, and according to Morrissey and Leyaro (2015), the system worked in a productive manner overall (albeit without dramatic results), especially that the state was consistent in establishing and supporting RTOs for clear goals, i.e., policymakers acknowledged the critical, multi-faceted role of RTOs in the national technology system and industrial development, and there was a political will standing behind their support. The acts of government that established RTOs (URT 1973; 1979; 1980; 1981; 1986) reflect that acknowledgement. With the advent of mixed economy policies, however, RTOs faced budget cut-downs and had to interact with broader industries (mostly private) according to market rules, where industries need persuasion to uptake new technologies and where direct government support is limited. Policies and practices of RTOs remained unchanged while Tanzania changed economic models, rendering a case of ‘command economy RTOs’ under a mixed economy state.

Key informant respondents were also invited to provide their perspectives on how RTOs could enhance their performance (i.e., suggestions). They were asked to provide suggestions based on micro, meso and macro levels (as in the analytical tool/policy map):

On improving the national enabling environment (macro level), respondents suggested that the acts of parliament governing RTOs (each and all) should be amended, because the current ones are outdated and seem to hinder broader possibilities.^9^ Participants also suggested that all RTOs should be managed under one umbrella, instead of running under various ministries as they are now. TEMDO, TIRDO and CAMARTEC could work together more closely, because they do the same thing essentially.^10^ Several respondents emphasized that national political commitment (political will) is a must.^11^ Additionally, increase of funding and financial resources is a major priority upon which the rest of the changes depend.^12^ For example, a respondent proposed that taxes on industrial imports can be used to fund RTOs, especially imports that have local equivalent products.^13^

On improving the network of RTOs (meso level), respondents suggested that RTOs should work together and communicate much more than currently so, and should also strengthen linkages with equivalent organizations in neighbouring countries, plus regional and international collaboration; for example, between Tanzanian and Kenyan RTOs.^14^ Another suggestion was to harmonize skills between RTOs and universities, so that there can be a horizontal two-way movement of qualified researchers between them. This will provide a good supply of researchers and research ideas for RTOs while provide university researchers with applied research projects of national interest to work on.^15^ A few respondents suggested that RTOs’ clients should be empowered by measures such as including private sector members in governing boards of RTOs.^16^

On enhancing organizational capacity (micro level), respondents suggested to improve HR capacity by hiring more skilled staff and upgrading skills of existing staff (with scholarships to pursue advanced studies with local research projects, etc., ) and giving them convincing packages to stay and continue.^17^ In addition, respondents suggested improving the management systems of RTOs to optimize use of human, capital and financial resources in projects.^18^ Another suggestion was to put forth direct regulations to increase practical programs such as incubators.^19^ Several respondents also suggested the improvement of infrastructure: finish unfinished buildings in RTO premises, renew machine shops with modern equipment, and make laboratories certified.^20^ A few respondents also suggested exploring innovative ways to make technologies of RTOs affordable to clients, such as rentals or group ownership and installment-payments for agricultural machineries for small farmers.^21^

### Brief Comparative Cases: Kenya and Malaysia

The study briefly explored the situation of relevant RTOs in both Kenya and Malaysia, for brief comparative purposes.^22^ The selection of these two countries was based on expressed interests of the Tanzanian government and Tanzanian RTOs in learning from the two countries: short-term for Kenya and long-term for Malaysia. Tanzania and Kenya share considerable aspects of their political-economic history as well as their policies of industrial development, since colonial times and the post-colonial East African Community years (Coulson 1982), yet they also diverge on several policies and circumstances that made Kenya’s current industrialization level, overall, ahead of Tanzania’s (Pietrobelli 2001; Lall and Pietrobelli 2005). As for Malaysia, it was selected as a benchmark—i.e., the country against which Tanzania is benchmarking progress toward industrialization—because the Tanzanian government itself declared Malaysia as a reference story for transforming from a low-income to middle-income, semi-industrialized country (FYDP-II 2016; [Commission for Science and Technology (COSTECH), 2015].^23^

The Kenya Industrial Research and Development Institute (KIRDI) has relatively more resources at its disposal as well as a larger number of highly qualified personnel in-house and a more conducive enabling environment. These advantages were correlated with more output to show for—more patents registered than any of the Tanzanian RTOs involved in this study, more research publications, and more graduated incubatees.^24^ According to KIRDI personnel, their staff overall publish an average of 50 publications annually, about 20 patents were filed, with one issued, and with plans to have them licensed to Kenyan industries.^25^ Through its own revenue-generating activities—such as consultancies, training programs, successful grant submissions, incubations and industry fellowships, mass production for industry clients, and future licensing of patents—KIRDI claims to be *en route* to becoming self-sustained by 2025. Lall and Pietrobelli (2005) attributed improvements to the operations of KIRDI—which was more or less in similar situation to Tanzania’s TIRDO in the 1990s—to changes that began in 1994: “In 1994, the findings of a United Kingdom team examining R&D institutions in Kenya led the government to reorient them to industrial needs. KIRDI was placed under a new director, who redefined its work to move from R&D to industrial technology support and reorganised the institution … ” (p. 332). According to 2010 innovation indicators,^26^ gross domestic government expenditure on R&D (GOVERD) in Kenya was 0.40% of the country’s GDP, with a total of 0.79% of GDP spent on R&D (GERD); while in Tanzania GOVERD was 0.07% of GDP and GERD was 0.53%. In Kenya, over 69% of R&D is funded by the state, while in Tanzania the same proportion is about 42%.^27^

In Malaysia, the Standards and Industrial Research Institute of Malaysia [Standards and Industrial Research Institute of Malaysia (SIRIM), 2016] stands out. It was established in 1975, and today its main campus contains about 25 buildings (including laboratories, assembly and testing facilities, as well as offices) with 2000 workers, and has several branches around the country. SIRIM was corporatized in 1996, but the Malaysian state continues to be its main source of revenue as a client of large-scale services such as establishing and monitoring industrial and product standards, conducting testing of products and procedures for quality control, providing certified quality training to workers on various key sectors, and conducting national industrial improvement projects sanctioned by the government [Standards and Industrial Research Institute of Malaysia (SIRIM), 2015]. SIRIM produces periodical reports to the Ministry of Science, Technology and Innovation and the Ministry of Finance, so it is still a parastatal. What is noticeable is that SIRIM conducts activities that are equitable to the overall activities of several Tanzanian RTOs (such as TIRDO, TEMDO and CAMARTEC) and more, such as establishing and maintaining national standards of products and industrial procedures, which are similar to activities conducted by other metrology institutions in Tanzania and Kenya.^28^ In other words, the large size of SIRIM, and the prolific profile it boasts, may be partly attributed to that it is both logistically and officially an equivalent of multiple RTOs in Tanzania. SIRIM is partly a result of keen investment that the Malaysian state placed in RTOs as part of its industrial policy. Rasiah and Shari (2001) and Lall (1995) highlight that Malaysia followed a broad and consistent industrial development policy between the 1970s and 1990s—‘New Economic Policy’—and that state investment in industrial R&D and commercial parastatals was part of that policy. A combination of state direct intervention, investment and incentives, as well as private sector support, drove Malaysia through a period of accelerated industrialization. SIRIM was given serious attention (indicating an appreciation of its multi-faceted role as an RTO) and that investment has produced fruit. For example, SIRIM’s Industrial Incubator Scheme (for SMEs), which started in 1986, “successfully graduated more than thirty incubatees (by 2000) in various technology areas (such as) design and metal fabrication, plastic moulding, chemical and industrial biotechnology and electronics (and) at least 60 percent of SIRIM incubator graduates (were) still in business.” (Yunos 2002, 186).

## Discussion and Lessons

Lessons learned from the key informants’ feedback and systemic literature review in this study confirm that the Tanzanian triple helix, industry-research policy areas and research-industry frontiers could use more proactive facilitators, i.e., intermediaries, to strengthen linkages between research, industry and government, to eke out more value and more technological supply to local demand: (i.e., technology localization). RTOs are currently in a peculiar place: they possess valuable technical skills, knowhow, infrastructure, institutional memory, and connections to urban and rural productive sectors. These assets would make them well-positioned to serve national technology systems and technological innovation processes as super intermediaries. Moreover, if ways can be found to alleviate institutional barriers and transform RTOs in a low-income country, the implications of such findings can feed into relevant and effective policy packages for that country as well as reach beyond one country to others with similar cases.

### If Policymakers Saw RTOs as Super Intermediaries

As mentioned in the findings, Tanzania’s national plans generally acknowledge RTOs as instrumental in bigger flagship projects and sectoral plans. For that reason, some of the case study respondents had good reasons to be optimistic about the FYDP-II, although the same national plans do not speak of revamping or improving the conditions of RTOs.

When they were established, RTOs had a strong political will behind them, making sure they function as expected and within a larger scheme. Policymakers at that time expressed a comprehensive appreciation of the key, multi-faceted role RTOs play in a country’s industrialization process. That appreciation was reflected in the acts of government that established these organizations. Currently RTOs seem to be often busy with survival, where they seek to secure sufficient resources for remaining relevant in the big picture. Tanzania invested in them previously, and at this phase of aspiration for industrialization they can bring good return on investment but only with additional investment. Given the current barriers, the potential of RTOs in Tanzania could not be unlocked if they continue with the status quo. In situations like these, things tend to continue in slow motion unless some interventions disrupt business as usual. Policy changes may trigger changes that snowball into bigger ones inclusive of national systems.

An argument that could be supported from historical evidence (in Tanzania and in countries where RTOs played key roles in industrialization, mentioned earlier) is that it is not enough for national policymakers to see RTOs as instrumental in bigger projects, but to see them as super intermediaries—i.e., playing a critical role in the big picture of multiple fronts: the triple helix, industrial-research policy overlaps, and research-industry frontiers. If seen as such, it would follow that they will be utilized as such (or given room to function as such) and it will also follow that investing in revamping RTOs draws from investing in the said fronts altogether. This change in perspective changes policy orientation and hence prioritization of investing in RTOs in themselves and not only as secondary participants in bigger projects. Therefore, it can be argued that the proposed conceptual framework is useful in this direction.

### Policy Reform Possibilities

Policy reform can be generally defined as ‘a process in which changes are made to the formal “rules of the game”—including laws, regulations and institutions—to address a problem or achieve a goal such as economic growth, environmental protection or poverty alleviation.’ [Organization for Economic Cooperation and Development (OECD), 2007]. Do Tanzanian RTOs need policy reform?

COSTECH is the main science granting council, and distributor of the state’s R&D allocated budget. They generally seem to be satisfied with their policy of competitive research funding, where research teams in Tanzania—from universities, RTOs, NGOs and private sector organizations—receive funding based on the quality of their proposals (or responses to calls) and their institutional and HR competency for carrying out projects. With this approach, COSTECH can report to the government that they channeled public funding to the most deserving recipients. COSTECH senior personnel expressed that the introduction of competitive-based funding is one of the good things that happened recently. Other RTOs have quite a different perspective. As expressed through a number of RTOs’ personnel involved in this study, they think competitive funding from COSTECH is a disadvantage.^29^ For these RTOs that have always depended mainly on state funding for research what COSTECH is doing now means that they will receive even less funding because they cannot compete with big universities or big companies, which either have or can afford more veteran researchers as well as relatively better laboratories. Under the current circumstances, RTOs feel punished for not having what they cannot have, i.e., more researchers and better infrastructure, precisely because they do not have the funding to change these conditions—a catch-22. This same predicament has been confirmed and expressed by multiple science granting councils across Africa through more recent studies, such as that by Hanlin, Tigabu and Sheikheldin (2021) and Chataway et al. (2019). This is a policy problem; particularly policies that govern the distribution of resources to RTOs and the mechanisms and criteria by which resources are received and used.

### Reconciling Reverse Engineering and IPRs

Historical experience tells that many countries in the second half of the 20th century took successful leaps towards industrialization through systemic reverse engineering efforts [Science, Technology and Innovation Policy Research Organization (STIPRO), 2010; Lall 1992]. RTOs in Tanzania are no strangers to such activities, but they could be enhanced.^30^ However, would there be contradiction between supporting both cultures of protecting IPRs (intellectual property rights)—through patenting—and reverse engineering? This question reflects a larger debate within the field, but the debate itself is more about relative dosages of both choices rather than ‘either-or’ absolute sides. Reconciliation options should be explored, i.e., selective dosages of cultures of technological adaptation and patenting. The two cultures should coexist in contexts of developing countries, to deal with both adapting long-existing technologies and coping with new/emerging technologies.

## Conclusions and Broad Recommendations

Based on existing records, historical investigation and informed opinions, particular constraints and policy aspects were identified that formed barriers to encouraging RTOs in Tanzania to fulfill their role as super intermediaries within the triple helix, industrial-research policy overlaps, and research-industry frontiers (Figure 1). Opportunities were explored in terms of policy reform. The findings were also generally in-line with the two main lenses of inquiry: 1) that, in order for RTOs to play their key roles in Tanzania, industrial policies shaped by the command economy era before the 1990s need to be reviewed and modified; and 2) that more investment in revamping RTOs will likely take place if policymaking processes acknowledged RTOs as key players on multiple fronts (i.e., super intermediaries).

As a normative synthesis to the study, some policy lessons are put forth in the form of broad recommendations, based on the macro, meso, and micro levels (Figure 2). They are not meant to be exhaustive or detailed, as they only try to harvest and capsulize the policy lessons learned, or highlighted, from this study.

On the macro level (national enabling environment), national policy should practice the established wisdom that, in order to reap reliable returns, additional investment is required in some strategic parts of the national technology system—i.e., to get more from their existing infrastructure and potential there should be more investment in empowering RTOs to do what they are meant to do. For example, laws governing RTOs can be amended to be in-line with the current macroeconomic reality (mixed economy) and current national development plans. Particularly RTOs should have clear mandate to establish and sustain revenue-generating activities within a non-profit framework. There should also be a commitment of specific annual public funds to RTOs, for applied research or R&D activities without the need to enter competition for such funds with other research institutions such as universities. RTOs could also be directed, *via* policy and funding incentives, to focus their R&D efforts on critical sectors, facilities and projects in-line with national flagship projects. Some RTOs can also establish a framework that allows them to ‘act as one’ in various projects and programs (including proposals, resources, standards, etc.,.).

On the meso level (among RTOs, Partners and Stakeholders), RTOs could be systematically and wholly orientated toward local industries. This will require big institutional shifts and a big process of network building. For example, local industry leaders, from the private sector, should have placements in RTOs’ governing boards. The main common tasks should be graduating SME incubatees, completing successful technical consultancies, and creating spin-offs that diffuse and commercialize the technologies born out of RTO’s R&D projects. And to increase the pool of human resources for RTOs, a policy of two-way human resource mobility between RTOs, universities and technical colleges could be implemented, for skilled personnel, so that there can be a safe horizontal movement for career researchers between these institutions. Furthermore, some good frameworks already exist but need to be activated. Such frameworks could become the main representative framework of the meso level among RTOs and local partners. On the micro-Level (within each RTO), rigorous management systems could be introduced (such as Results Based Management, and Monitoring, Evaluation and Learning systems). As entities supported by public funds, it is important to subject RTOs to periodic comprehensive evaluations, with the mandate to fulfill minimum requirements of standards and outputs to justify continuous public funding.

One of the main limitations of this study is that it proposes a conceptual framework whose usefulness should be further tested, by other researchers as well as the author, since proving relevant for one case study is not enough. Further studies could either examine more in-depth cases using the framework or explore whether RTOs comprehensively, in various countries and regions, fulfill the super-intermediary role, or do so in only limited contexts.

## Data Availability

The original contributions presented in the study are included in the article/Supplementary Material, further inquiries can be directed to the corresponding author.

## Footnotes

1 According to WAITRO (World Association of Industrial and Technological Research Organizations), the most recognized global umbrella for such organizations (and part of the United Nations apparatus), the acronym RTOs covers a wide but well-defined spectrum. They have also been called ‘industrial support organizations’, ‘public technology intermediaries’, ‘R&D parastatals’, etc.

2 See Sheikheldin (2015); Sheikheldin and Devlin (2019) in references.

3 Science granting councils (SGCs) are public institutions that often fund science and technology research, in addition to managing and coordinating such research (for more info, Hanlin et al., 2018).

4 According to COSTECH’s leading personnel we interviewed for this project.

5 Interviews were not recorded (audio or video). Responses were written down during each interview. This way of recording answers was deemed suitable for this research, through the research ethics approval process completed with REB of the University of Guelph, and has the advantage of making interviewees more relaxed and interactive in the interview. Consent forms were signed by all interviewees and/or their organizations.

6 Interview: XD07

7 Interview: XB03

8 Interviews: EA45, BD17, AA07 and others

9 Interviews: DD33, AA07, XC05, CC20, BA15, BE12 and others

10 Interviews: ED49, XD07

11 Interviews: CB24, BB13, BD17, CD25

12 Interviews: DA35, AB04, AD09, XC05, EA45, EB44, BA15, BB13, BD17

13 Interview: XB03

14 Interviews: XE09, AA07, CA26

15 Interviews: XE09, DD37

16 Interviews: AA07, CB24

17 Interviews: DA35, BC19

18 Interview: EA45

19 Interview: AB04

20 Interviews: XE09, DA35, BA15, CD25

21 Interviews: DA35, DB34

22 Additionally, the researcher did not have the proper research clearance or the funds to systemically gather data from Kenya or Malaysia, and so only scouted the countries, met relevant people and gathered relevant public information within the limits allowed, legally and ethically, in a brief exploring tour.

23 Malaysia’s story of national development is expected to guide many policies and implementations that Tanzania will consider seriously for its own on-going story. For example, ‘FYDP I initiated the Big Results Now (BRN) as one of its tools for (Monitoring and Evaluation), particularly for tracking and ensuring implementation in designated key result areas (KRAs). BRN was inspired by Malaysia’s Big Fast Results approach.

24 Interview: FA88

25 Numbers were not verified through official records or independent sources.

26 Sources: NEPAD. 2010 and 2014 (April). African Innovation Outlook (I and II); and World Bank Data; most recent available data.

27 It is noted, however, that most of Tanzania’s expenditure on R&D went to higher education, significantly more than Kenya (Source: NEPAD, 2010 and National Commission for Science, Technology and Innovation, Kenya (NACOSTI), 2014).

28 Interview: in-person interview with senior manager, SIRIM Industrial Research, Kuala Lumpur.

29 Interviews: BD17, EA45, DD37 and others

30 Interviews: ED49, DB34, DA35 and others.
