# Supplementary material for: Research and Technology Organizations as Super Intermediaries: A Conceptual Framework for Policy and a Case Study From Tanzania
Source: Front Res Metr Anal. 2021 Jun 17;6:691247. doi: 10.3389/frma.2021.691247 (PMC8245844; doi:10.3389/frma.2021.691247)
Supplement: Supplementary file 1 [file Table1.DOCX]

# **Appendices**

***Appendix I: List of interviews***

(Names were replaced by with codes, and identifying information removed for confidentiality)

| **Code** | **Association** | **Interview Date** | **Relevant Experience** |
| --- | --- | --- | --- |
| **AA07** | COSTECH | 21/4/2016 | 5-15 years, graduate degree |
| **AB04** | COSTECH | 22/4/2015 |  |
| **AC06** | COSTECH | 22/4/2016 | +15, graduate deg. |
| **AD09** | COSTECH | 25/4/2016 | 0-5 years, graduate deg. |
| **AE01** | COSTECH | 29/4/2016 | 5-15 years, graduate deg. |
| **BA15** | TIRDO | 5/5/2016 | +15 years, graduate deg. |
| **BB13** | TIRDO | 5/5/2016 | 5-10 years, graduate deg. |
| **BC19** | TIRDO | 5/5/2016 | 0-5 years, graduate deg. |
| **BD17** | TIRDO | 10/5/2016 | +15 years, graduate deg. |
| **BE12** | TIRDO | 10/5/2016 | graduate deg. |
| **CA26** | SIDO | 10/5/2016 |  |
| **CB24** | SIDO | 11/5/2016 | +15 years, graduate deg. |
| **CC20** | SIDO | 11/5/2017 | +15 years, undergraduate deg. |
| **CD25** | SIDO | 11/5/2018 | 5-15 years, undergrad deg. |
| **XA01** | UNIDO Tanzania | 11/5/2019 |  |
| **XB03** | UNIDO Tanzania | 11/5/2020 | Engineering Background. |
| **XC05** | Ministry of Education, Science & Technology | 12/5/2016 | 5-15 years, graduate deg. |
| **XD07** | Ministry of Industry and Trade | 13/5/2016 |  |
| **XE09** | University of Dar es Salaam | 13/5/2016 | +15 years, graduate deg. |
| **XF11** | STIPRO (Science, Technology & Innovation Policy Research Organization) | 17/5/2016 | 5-15 years, graduate deg. |
| **DA35** | CAMARTEC | 19/5/2016 | 5-15 years, graduate deg. |
| **DB34** | CAMARTEC | 19/5/2016 | 5-15 years, undergrad deg. |
| **DC37** | CAMARTEC | 19/5/2016 | 0-5 years, graduate deg. |
| **DD33** | CAMARTEC | 20/5/2016 | +15 years, graduate deg. |
| **DE38** | CAMARTEC | 20/5/2016 | 0-5 years, undergrad deg. |
| **EA45** | TEMDO | 23/5/2016 | +15 years, graduate deg. |
| **EB44** | TEMDO | 23/5/2016 | 5-15 years, graduate deg. |
| **EC41** | TEMDO | 23/5/2016 | 0-5 years, graduate deg. |
| **ED49** | TEMDO | 23/5/2016 | 5-15 years, undergrad deg. |
| **EE48** | TEMDO | 23/5/2016 | 5-15 years, undergrad deg. |
| **FA88** | KIRDI | 8/6/2016 | Kenya's equivalent of TIRDO |
| **FB66** | NACOSTI | 8/6/2016 | Kenya's equivalent of COSTECH |
| **FC55** | ACTS (African Centre for Technology Studies) | 9/6/2016 | Pan-African intergovernmental organization, a think tank for technology research. |
| **FD11** | The Scinnovent Centre | 10/6/2016 | Research and consultancy NGO, Kenya |
